# Transmission experiments verify sporadic V2 prion in a patient with E200K mutation

**DOI:** 10.1007/s00401-024-02738-6

**Published:** 2024-05-20

**Authors:** Hitaru Kishida, Atsushi Kobayashi, Kenta Teruya, Hiroshi Doi, Naohisa Ueda, Fumiaki Tanaka, Yoshiyuki Kuroiwa, Piero Parchi, Shirou Mohri, Tetsuyuki Kitamoto

**Affiliations:** 1 Department of Neurology, Yokohama City University Medical Center, Yokohama, Japan; 2 Department of Biomedical Models, Graduate School of Biomedical Sciences, Nagasaki University, Nagasaki, Japan; 3 Department of Neurochemistry, Tohoku University Graduate School of Medicine, Sendai, Japan; 4 Department of Neurology and Stroke Medicine, Yokohama City University Graduate School of Medicine, Yokohama, Japan; 5 Department of Neurology, Teikyo University School of Medicine University Hospital, Kawasaki, Japan; 6 Department of Biomedical and Neuromotor Sciences, University of Bologna, Bologna, Italy; 7 Istituto Delle Scienze Neurologiche Di Bologna (ISNB), IRCCS, Bologna, Italy; 8 Office for Research Initiatives and Development, Nagasaki University, Japan Nagasaki,; 9 Department of Neurological Science, Tohoku University Graduate School of Medicine, 2-1, Seiryo-machi, Aoba-ku, Sendai, Miyagi-ken 980-8575 Japan

Creutzfeldt-Jakob disease (CJD) is caused by abnormal pathogenic prion protein (PrP^Sc^), which is generated by conformational change of normal cellular isoform (PrP^C^) [6]. The conversion occurs due to either one of three causes: spontaneous conversion in sporadic CJD (sCJD), conversion triggered by pathogenic mutation of the prion protein gene (*PRNP*) in genetic CJD (gCJD), or infection with PrP^Sc^ in acquired CJD. In sCJD, there are six subtypes based on two factors: (1) the genotype at polymorphic codon 129 of the *PRNP* gene (Methionine, 129M or Valine, 129V), and (2) the PrP^Sc^ type in brain tissue, which are distinguishable according to the size of protease-resistant core in Western blot analysis (21 kDa, type 1 or 19 kDa, type 2) [5]. The E200K mutation is the most common *PRNP* variant worldwide in gCJD. The clinicopathologic features of most gCJD patients with the E200K mutation are pretty similar to those of typical sCJD, *i.e.*, sCJD-MM1/MV1, hence named as the M1 subtype [1]. In the M1 subtype, the E200K mutation is present on the 129M allele, and type 1 PrP^Sc^ is detected in the brain tissue [4]. However, some patients with the E200K mutation on the 129V allele show type 2 PrP^Sc^ accumulation and manifest a clinical course similar to sCJD-VV2; hence, they have been classified as the V2 subtype [1, 2]. Finally, a few patients carrying the E200K-129M haplotype showed clinicopathologic features of the V2 subtype in association with a PrP^Sc^ type of intermediate size between types 1 and 2 [1]. We report for the first time the transmission properties of a CJD patient carrying the E200K-129M haplogype with clinicopathologic features of the V2 subtype.

A 61-year-old Japanese woman presented with dizziness and ataxia, cognitive decline 4 months later, and further myoclonus, pyramidal tract signs and extrapyramidal symptoms, which progressed to akinetic mutism and death 12 months later. Brain MRI diffusion-weighted images showed hyperintensities in bilateral basal ganglia, and no periodic synchronous wave complexes were found in the electroencephalogram (EEG). *PRNP* genetic test revealed the E200K mutation and 129M/V heterozygosity (Fig 1a). Clinically, the patient was tentatively diagnosed as gCJD-E200K. However, the clinical features of this case resembled those of sCJD-MV2 rather than the M1 subtype of gCJD-E200K. She had no history of acquired prion disease.

**Fig. 1:**
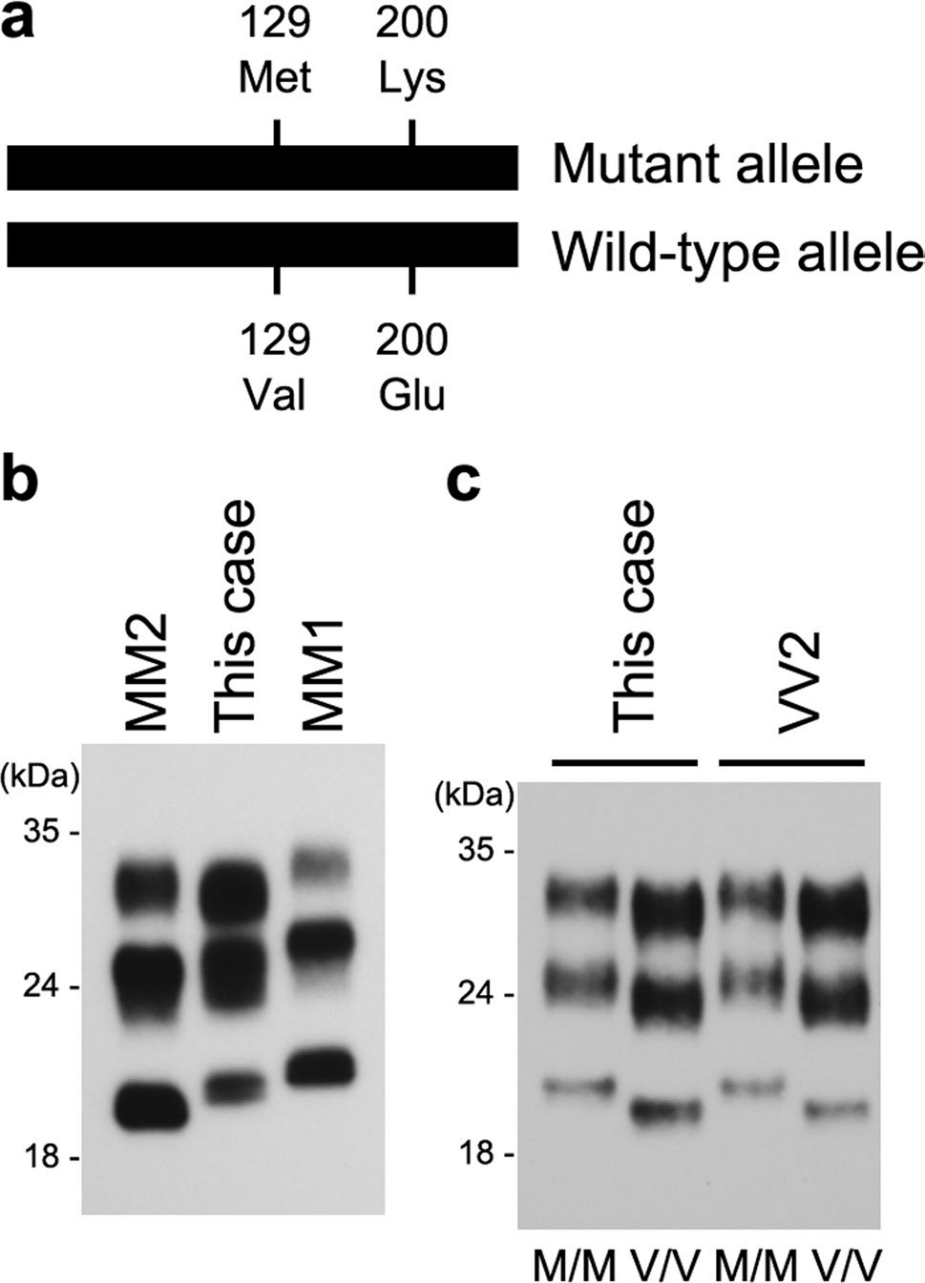
Biochemical properties and transmission properties of PrP^Sc^ from the patient with the E200K mutation and the 129M/V heterozygosity. **a** The genotypes at codon 200 and a polymorphic codon 129 of the *PRNP* gene. The patient carried 200K-129M haplotype and 200E-129V haplotype. **b** Western blot analysis of protease-resistant PrP^Sc^ in the patient’s brain. The patient had the intermediate type PrP^Sc^ located between type 1 (sCJD-MM1) and type 2 (sCJD-MM2) PrP^Sc^. **c** Western blot analysis of protease-resistant PrP^Sc^ in the brains of the inoculated PrP-humanized knock-in mice. Ki-129M/M mice (M/M) produced the intermediate type PrP^Sc^, while Ki-129V/V mice (V/V) produced type 2 PrP^Sc^ when inoculated with the brain material from the patient. These transmission properties were the same as those of sCJD-VV2

Brain weight was 1180 g, and the cerebral cortex and the subcortical white matter were macroscopically well preserved. Histologically, the cerebral cortex showed typical spongiform changes in the deep gray matter. However, the number of neurons was well preserved, gliosis was mild, and granular cells were preserved in the cerebellum. In the cerebellum, there was a marked decrease in the number of cells in the dentate nucleus, resulting in the loss of nerve fibers in the superior cerebellar peduncle. The inferior olivary nucleus also showed moderate cell count depletion. PrP-immunostaining showed mainly synaptic-type deposits in both the cerebral cortex and cerebellum, with prominent perineuronal deposits in the cerebral cortex, and small patchy paler accumulations in parts of the cerebral cortex and in the molecular layer of the cerebellum. The cerebellum did not show typical kuru plaques. These pathological findings were similar to those of the V2 subtype.

Western blot analysis of PrP^Sc^ was performed using conventional anti-PrP antibodies (3F4, type 1 PrP^Sc^-specific antibody T1, or type 2 PrP^Sc^-specific antibody T2). In this case, unglycosylated PrP^Sc^ was detected at 20 kDa using the 3F4 antibody (Fig. 1b), and a very small amount of band was detected at 19 kDa using the T2 antibody (data not shown). These findings indicate a mixture of predominant intermediate-type PrP^Sc^ and a tiny amount of type 2 PrP^Sc^.

Next, we performed transmission experiments to confirm the presence of the V2 prion and the absence of the M1 prion. Brain homogenates of this case and a sCJD-VV2 case were inoculated into PrP-humanized knock-in mice expressing human PrP^C^ with the 129M/M (Ki-Hu129M/M) or 129V/V genotype (Ki-Hu129V/V) and knock-in mice expressing human-mouse chimeric PrP^C^ (Ki-ChM) [7]. The inoculated Ki-Hu129V/V showed a shorter incubation period than the Ki-Hu129M/M (Table 1). The inoculated Ki-ChM did not develop prion disease despite Ki-ChM being highly susceptible to the M1 prion strain. In Western blot analysis of protease-resistant PrP^Sc^, Ki-Hu129M/M produced the intermediate-type PrP^Sc^, while Ki-Hu129V/V produced type 2 PrP^Sc^ (Fig 1c). These findings indicated that this case harbored only the V2 prion and not the M1 prion.

**Table 1:** Transmission to PrP-humanized knock-in mice

Inoculated material	Attack rate^a^ (mean incubation period, days)		
	Ki-129M/M	Ki-129V/V	Ki-ChM
This case	4/4 (691 ± 30)	5/5 (422 ± 26)	0/8
VV2	5/5 (670 ± 79)	6/6 (313 ± 4)	0/5

^a^Number of mice positive for PrP^Sc^ accumulation/number of inoculated mice

This patient had the E200K mutation and the 129M/V genotype (200K-129M / 200E-129V haplotypes); the clinical features were similar to those of sCJD-MV2, the neuropathologic features resembled those of the V2 subtype, and the brain contained the intermediate-type PrP^Sc^ and a small amount of type 2 PrP^Sc^. The intermediate-type PrP^Sc^ is seen in dura mater-graft associated CJD-129M [8], sCJD-MV2 [3], and in a rare subgroup of gCJD 200E-129M individuals, and the V2 prion is thought to convert the 129M PrP^C^ into the intermediate-type PrP^Sc^. Indeed, the transmission properties of the present case were compatible with those of the V2 prion strain. Thus, these findings suggest that, either the mutated PrP with 129M generated the V2 prion conformation or there was a spontaneous formation of the V2 prion from the wild-type 129V allele, which subsequently converted the mutant 129M PrP^C^ into the intermediate-type PrP^Sc^. The absence of the V2 prion in gCJD-E200K patients with the 129M/M genotype may lend support to the latter hypothesis.

The sporadic V2 prion disease in a patient with the E200K mutation, as in this case, may not be rare. In a previous study of a large gCJD patient cohort, there were 18 cases of gCJD-E200K with the 129M/V heterozygosity [1]. Among them, 16 cases had the 200K-129M/200E-129V haplotypes, eight of which showed the intermediate-type PrP^Sc^ and clinicopathological features of the V2 subtype as with the present case. The other five and three cases were the M1 and M2C subtypes, respectively. Thus, half of the reported gCJD-E200K cases with the 200K-129M/200E-129V haplotypes might represent sporadic V2 prion disease. These findings suggest that caution is required in data analysis of gCJD-E200K in clinical, diagnostic, and therapeutic studies.

## Data Availability

All data supporting the findings of this study are available from the corresponding author upon request.
